# Breast cancer: patient information needs reflected in English and German web sites

**DOI:** 10.1038/sj.bjc.6602168

**Published:** 2004-10-05

**Authors:** C Weissenberger, S Jonassen, J Beranek-Chiu, M Neumann, D Müller, S Bartelt, S Schulz, J S Mönting, K Henne, G Gitsch, G Witucki

**Affiliations:** 1 Division of Radiotherapy, University Hospital of Freiburg, Hugstetter Strasse 55, D-79106 Freiburg, Germany; 2 Institute of Medical Biometry and Medical Informatics, University of Freiburg, Department of Medical Informatics, Stefan-Meier-Strasse 26, D-79104 Freiburg, Germany; 3 Institute of Medical Biometry and Medical Informatics, Department of Medical Biometry and Statistics, University of Freiburg, Stefan-Meier-Strasse 26, D-79104 Freiburg, Germany; 4 Department of Obstetrics and Gynecology, University Hospital of Freiburg, Hugstetter Strasse 55, D-79106 Freiburg, Germany; 5 Department of Radiotherapy, Deaconess Hospital, Am Mutterhaus 1, D-74523 Schwäbisch-Hall, Germany

**Keywords:** breast neoplasms, internet, quality assurance, health care, information services/^*^standards/utilisation, internet/^*^standards/utilisation, complementary therapies

## Abstract

Individual belief and knowledge about cancer were shown to influence coping and compliance of patients. Supposing that the Internet information both has impact on patients and reflects patients' information needs, breast cancer web sites in English and German language were evaluated to assess the information quality and were compared with each other to identify intercultural differences. Search engines returned 10 616 hits related to breast cancer. Of these, 4590 relevant hits were analysed. In all, 1888 web pages belonged to 132 English-language web sites and 2702 to 65 German-language web sites. Results showed that palliative therapy (4.5 *vs* 16.7%; *P*=0.004), alternative medicine (18.2 *vs* 46.2%; *P*<0.001), and disease-related information (prognosis, cancer aftercare, self-help groups, and epidemiology) were significantly more often found on German-language web sites. Therapy-related information (including the side effects of therapy and new studies) was significantly more often given by English-language web sites: for example, details about surgery, chemotherapy, radiotherapy, hormone therapy, immune therapy, and stem cell transplantation. In conclusion, our results have implications for patient education by physicians and may help to improve patient support by tailoring information, considering the weak points in information provision by web sites and intercultural differences in patient needs.

In December 2000, 42% of the European females were using the Internet with a clear upward trend (Kögel and Longo, 2001). Notably, women older than 50 years turned out to be the most active group of Internet users (Pichler and Gilg, 2001). In April 2001, Harris Interactive published results indicating that 75% of all adults who had access to the Internet used the Internet to collect health and medical information (Taylor and Leitman, 2001). In the USA, 54% of the Internet users searched the Web for health-related information compared to 26% in Europe, as demonstrated by a survey of Health On the Net (HON) Foundation in 2001. More females (51%) than males (49%) used the Internet to search for health-related information (HON, 2002). The main obstacle for patients is ‘that there is so much information on the Net, big parts of it are incomplete, misleading, or inaccurate’ (Achenbach, 1996). Correspondingly, Silberg *et al* (1997) had criticised the information on the Internet as not peer reviewed and pointed out the dangers of relying on such invalidated information.

Due to the high incidence of mamma carcinoma (Weir *et al*, 2003), breast cancer web sites attract a high number of information seekers. In addition, breast cancer patients have gained a higher awareness of their disease during the last decades. They demand breaking taboos considering breast cancer, they refuse to hide their illnesses any longer, and many develop a different attitude towards their disease, which is known as ‘fighting spirit’ (Greer, 2000). Breast cancer patients, therefore, can be considered pioneers of a new type of self-confident patients (Biel, 1997).

Well-informed patients often scrutinise doctors' recommendations, search the Internet for specialists, and contact other patients all over the world (Degner *et al*, 1997; Blanchard *et al*, 2002). These patients want to participate actively in the therapeutic decisions and use the Internet ‘to acquire expertise to display competence in the face of serious illness’ (Ziebland *et al*, 2004). But, as Internet users, they play both an active and passive role. The passive role explains why these patients might be influenced by Internet information, and the active role – for example, patients as web authors – explains why the Internet reflects patients' needs. Both roles contribute to patients' individual belief and knowledge about cancer, which was shown to influence their coping and compliance.

But very little is known about intercultural differences between breast cancer patients (Nicholson, 1996; Smith *et al*, 2001) and, even less, about differences between English- and German-language web sites as mirrors of intercultural differences in patients' needs. Though the Internet is propagated as a ‘global medium’ theoretically enabling millions of people worldwide to communicate and share information electronically, the Internet as used by cancer patients emerged as a ‘local medium’, limited by language barriers. Analysing the internet use of 927 patients with breast cancer, we found that 260 (28.1%) among these are using the Internet to obtain information about their disease (unpublished data). More than a third of these patients with breast cancer (35.0%) turned out to be monolingual. Therefore, an increasing number of internet users are unable to use any foreign language web information autonomously. Conversely, their contributions to the Internet are inaccessible to monolingual members of any foreign language audience. This evokes questions about intercultural differences in disease management, pharmaceutical markets, and patients' behaviour and needs, mirrored by the Internet. Therefore, we analysed the Internet by evaluating a substantial number of breast cancer web sites in English and German language, representing the most and second-most frequent language (52.0 and 7.0%, respectively) used on the Internet (Pimienta *et al*, 2001).

## MATERIALS AND METHODS

### Search techniques to collect web sites for analysis

For evaluation purposes, a meta-search procedure was established and validated at the University Hospital in Freiburg, Department of Radiotherapy. To search for the keywords ‘breast cancer’, ‘Brustkrebs’ and ‘Mammakarzinom’, we modified (‘patched’) the meta-search programme Copernic^©^ 5.0 (Kunst *et al*, 2002) to enable the programme to download up to 10 000 hits from 18 popular search engines (instead of the default limitation to 100 hits), representing the pooled content of about 1000 search engine result pages. Installed on local computers, Copernic^©^ 5.0 provided a common interface to download, filter, and group web search results and check for duplicates.

Between March 2001 and February 2004, the web sites included in the study were repeatedly visited. The web sites were evaluated firstly between March 2001 and November 2001 and secondly between November 2003 and February 2004, to confirm the results and to prove the persistence of the web sites. The first evaluation was immediately followed by a second evaluation carried out by an independent observer to minimise the inter-rater variability. According to the study protocol, all web sites with a Cohen's kappa of less than 0.7 (thus proving a high interevaluator variability) were excluded, modifying the protocol of Bichakjian *et al* (2002). Finally, only those web sites which were accessible for at least 2 years and disseminated medical information pertaining to breast cancer were included in the analysis. Irrelevant web pages were excluded, as were link pages, non-English- or non-German web sites.

### Definitions of the analysed subjects

Before the study started, the four observers (radiooncologist, gynecologist, and two researchers) received an identical training concerning the inclusion and exclusion criteria of the web sites to ensure the best possible data quality. Their training involved general aspects of computer and internet use, web search and programme-specific techniques, as well as education in breast cancer treatment, based on principles of evidence-based medicine, current guidelines, and consensus recommendations. Moreover, the observers were trained in judging web sites using the criteria and definitions of the study.

If a certain hit, which the search engines returned for the query, fulfilled the inclusion criteria, analysis was continued using an algorithm to select and combine the corresponding hits to ‘web projects’. The ‘extension’ of a web site was determined by locating the home page (where possible), and then exploring the web site by following the links to find all associated, lower hierarchical web pages. This procedure was used to identify all matching sites belonging to one ‘conclusive’ informational project. These complete web sites were called ‘web projects’ so as to avoid misunderstandings, because the term ‘web site’ lacked a generally accepted, content-related definition at the time of our study. The definitions used in our study are summarised in Table 1

**Table 1:** Definitions of web-technical terms used in this study

**Item**	**Definition**
Hyperlinks	Clickable content on a web page usually leads to another page, another site or another part of the same page
Hit	Results that a search engine returns for a specific query
Search engine	A tool for finding information on the Internet
Web page	Document designed for viewing in a web browser
Web site	A place on the Internet that is comprised of files (text or graphics) organised into a hierarchy
Web project	A closed informational entity on the Internet

.

### Approaching the web sites for evaluation

Primarily, the hyperlinks provided by the search engines were checked for relevance and categorised into ‘relevant’, ‘semirelevant’, and ‘nonrelevant’ according to Su *et al* (1998). Hyperlinks were considered ‘relevant’ if they offered information on several aspects of the disease regardless of the quality of the information provided. They were rated ‘partly relevant’ if they only dealt with one special aspect (e.g. web sites dealing with cancer prevention, aftercare, or mammography), and ‘irrelevant’ if they did not deal with any aspect at all (e.g. web sites reporting celebrities' battle with breast cancer). Nonrelevant web sites were excluded from the evaluation. The in-depth analysis was performed using items according to pre-defined criteria. The questionnaire used for the evaluation consisted of two parts: the ‘formal’ and the ‘content-related’ analysis – as we were aware of the limitation that no distinct differentiation between formal and content-related aspects is possible in nature. The formal analysis was based on Silberg's essential accountability criteria (Silberg *et al*, 1997) and included: *site disclosure* (ownership, advertising, commercial funding arrangements or potential conflicts of interest), *site currency* (dates of posting and updating), *site authorship* (authors and contributors, their affiliations and relevant credentials), and *site attribution* (references and sources). As described below, Silberg's criteria were applied in an extended version.

The ‘content-related’ aspects were evaluated by the in-depth analysis of the information provided by the web site using principles of evidence-based medicine, current guidelines, or consensus recommendations for the treatment of breast cancer.

### Questionnaire Part I: Silberg's criteria

The first part of the analysis used Silberg's validated criteria list extended by further items abstracting the quintessence of other published criteria for information quality (Table 2

**Table 2:** List of initiatives to assess and improve quality of information

**Designation**
Discern (Charnock *et al*, 1999)
HON (Code of Conduct) (HON, 2001)
Quality criteria of Electronic Publication in Medicine: Criteria Catalogue of GMDS-AG CBT (Schulz *et al*, 2001)
Health Web site Standards of URAC – American Accreditation Healthcare Commission (2002)
Quality criteria of NOAH (New York Online Access to Health) (NOAH, 2002)
OMNI-Guidelines for Ressource Evaluation (OMNI=UK's Gateway to High Quality Internet Ressources) (OMNI, 2002)
AGREE Instrument (AGREE=Appraisal of Guidelines Research and Evaluation) (Cluzeau and Burgers, 2002)
ARGUS Clearinghouse Rating Systems (Argus Associates, 2001)
Tips on Evaluation Web Resources (National Network of Libraries in Medicine) (McKenzie, 1996)
The Internet Guide to Construction of Quality Online Resources (Ciolek and Goltz, 2001)
The Concerted Action on Health Information Systems (Aktionsforum Gesundheitsinformationssysteme AFGIS) (Prümel *et al*, 2002)

). Silberg's criteria were rated as available or not. Thus, we additionally searched the web projects for aspects such as privacy protection, disclaimer to the links, usage of the presented information (e.g. the warning not to use the medical advice instead of contacting a physician), presence of a quality seal (HON, 1997), orthography, and grammatical errors. Further, a small size of each web page (measured in kbytes), leading to a fast loading time, is one important aspect of good web site usability. A maximum page size of around 40 kbytes is commonly recommended for quick access to the web site.

The up-to-dateness of the web sites was assessed by repeated visits in order to check their content for currency and modifications. This procedure documented modifications and aimed to uncover sites which automatically set the current date as the ‘last update’, thus leading the Internet user to believe that the site is current and state of the art.

### Questionnaire Part II: content-related analysis

The second part of the questionnaire analysed the presented information such as treatment options, alternative treatment options, screening, follow-up, treatment-related risks, side effects, treatment time, and information about self-help and support groups. The items of the validated questionnaire were analysed as per the protocol providing detailed instructions for content evaluation.

Statistical analysis was performed using SAS and JMP® (SAS Corp.). The data were evaluated with descriptive statistics, *χ*^2^ tests, and Cohen's kappa to ensure low inter-rater variability (Cohen, 1960).

## RESULTS

### Hits returned to the web query

Search engines returned totally 10 616 unique hits related to breast cancer (Table 3

**Table 3:** Number of hits contributed by each search engine

**Search engines**	**Breast cancer**	**Brustkrebs**	**Mammakarzinom**
Lycos	3170	4000	510
Fast Search	2500	2270	999
Hotbot	1035	996	278
Altavista	1000	1000	798
Excite	1000	53	31
Webcrawler	1000	54	2
CompuServe	980	941	n/a
Google	861	535	567
Looksmart	785	0	0
AOL	625	0	0
Open Directory Project	505	0	1
EuroSeek	500	500	500
Netscape Netcenter	500	6	1
Yahoo!	340	0	0
MSN	192	195	193
NBCi	134	135	135
Direct Hit	12	14	18
Goto.com	0	0	138
			
Total	15 139	10 699	4171
			
Doublets removed	8335	11 058^a^	
Unique hits provided by the search engines	6804	3812^a^	
Relevant web pages included in the study	1888	2702	
			
Corresponding web projects	132	65	

All search engines results pages were pooled and the doublets were removed (‘n/a’: not available).

a Hits returned for ‘Brustkrebs’ and ‘Mammakarzinom’ were pooled before further analysis.

). Of these, 6026 nonrelevant hits were excluded from the study, and 4590 relevant hits were analysed. Of these, 1888 web pages belonged to 132 English-language web sites and 2702 belonged to 65 German-language web sites. Correspondingly, English-language web projects consisted on average 14.3 web pages, whereas German-language web projects consisted on average of 41.6 web pages. In all, 13.7% (937 of 6804) of the English-language and 8.6% (328 of 3812) of the German-language web pages turned out to be semirelevant ones, providing only information about the partial aspects of breast cancer (mostly prevention or aftercare of breast cancer). No web site disappeared in the period of the 2 years of the study or was substantially modified. After the training period in the pre-study, the two independent raters achieved a kappa value of more than 0.7 for all web sites. Thus, all 197 web projects were included in the study.

In all, 81.2% (*n*=160) of the English- and German-language web projects targeted patients, whereas 5.1% (*n*=10) targeted physicians. Altogether, 6.6% (*n*=13) of the web projects targeted both patients and doctors, 6.6% (*n*=13) other groups (e.g. web sites only for patients' relatives or business-to-business communication sites).

### Formal aspects

To analyse the formal criteria, we used Silberg's core criteria (Silberg *et al*, 1997).

(1) *Authorship:* We identified the author(s) and their qualifications significantly more often on German-language web projects than on English-language web projects (Table 4

**Table 4:** Results of the content-related analysis (Chi-square test)

	**Web projects in**		
	**English**	**German**	***P*-value**
*Formal criteria*			
Author(s) mentioned^*^	40 (30.3%)	36 (55.4%)	<0.001
Qualification(s) of the author(s) mentioned^*^	30 (22.7%)	32 (49.2%)	<0.001
Referred literature	23 (17.4%)	7 (10.8%)	0.222
Contact E-mail given^*^	107 (81.1%)	44 (67.7%)	0.037
Privacy statement	31 (23.5%)	9 (13.8%)	0.141
Disclaimer^*^	53 (40.2%)	9 (13.8%)	<0.001
Advertising	80 (60.6%)	45 (69.2%)	0.237
Correct information on ‘last update’^*^	34 (25.8%)	35 (53.8%)	<0.001
Automatic update script	7 (5.3%)	2 (3.1%)	0.482
Last update <1 year^*^	102 (77.3%)	34 (52.3%)	<0.001
Last update <2 years^*^	18 (13.6%)	19 (29.2%)	0.008
Last update <3 years^*^	3 (2.3%)	8 (12.3%)	0.004
Last update >3 years	9 (6.8%)	4 (6.2%)	0.860

*Information on therapy options*			
Chemotherapy^*^	129 (97.7%)	47 (72.3%)	<0.001
Hormone therapy^*^	129 (97.7%)	43 (66.2%)	<0.001
Radiotherapy^*^	125 (94.7%)	45 (69.2%)	<0.001
Breast-conserving surgery	109 (82.6%)	49 (75.4%)	0.234
Immune therapy	21 (15.9%)	8 (12.3%)	0.502
Palliative therapy^*^	6 (4.5%)	22 (16.7%)	0.004
Stem cell transplantation^*^	22 (16.7%)	4 (6.2%)	0.040
Pain therapy	3 (2.3%)	3 (4.6%)	0.368
Alternative or complementary medicine^*^	24 (18.2%)	30 (46.2%)	<0.001

*Therapy-related information*			
Therapy time	55 (41.7%)	22 (33.8%)	0.290
Side effects^*^	77 (58.3%)	22 (33.8%)	0.001
Risks of therapy^*^	72 (54.5%)	22 (33.8%)	0.006
Current studies^*^	24 (18.2%)	2 (3.1%)	0.003

*Disease-related information*			
Cancer aftercare^*^	34 (25.8%)	27 (41.5%)	0.024
Risk factors^*^	15 (11.4%)	24 (36.9%)	<0.001
Prognosis	34 (25.8%)	39 (29.5%)	0.605
Genetics	43 (32.6%)	28 (43.1%)	0.149
Epidemiology	64 (48.5%)	36 (55.4%)	0.312
Self-help groups^*^	51 (38.6%)	35 (53.8%)	0.043

Significant results marked by asterisk.

).

(2) *Attribution:* Conversely, we found references, contact e-mail address, and information about privacy protection of the users more often on English-language web sites. A disclaimer, which warned that the site should not be used as a substitute for doctor's visit, was displayed in 40.2% of the English-language web projects compared to 13.8% (*P*<0.001) of the German-language web projects. In all, 14.0% of all English- and German-language web projects displayed the HON Seal (HON, 2002). More than 50% of these web projects had a commercial background. Only a few web projects had a remarkable accumulation of mistakes in orthography or grammar. Using an access with analogue transmission technique, the average download time of pages belonging to English web projects was 12.5 s (German web projects: 9.0 s). The average size of the English web pages was 70.4 kbytes (German web pages: 52.9 kbytes).

(3) *Disclosure:* Overall, information about the site owner could be obtained in 183 (92.9%) web projects. Web projects were provided by institutions such as universities or noncommercial organisations in 47.2% (*n*=92), industry, companies or commercial organisations in 41.6% (*n*=82), and private persons in 10.7% (*n*=21). Elements of advertising (e.g. banners, pop-ups) were detected on 80 (60.6%) and 45 (69.2%) of English- and German-language web projects, respectively (Table 4).

(4) *Currency:* According to the update information displayed, English-language web projects were significantly more frequently updated within the last 12 months than German-language web projects (Table 4). As revealed by repeated visits, the displayed information about updating was, in fact, accompanied by an observable modification of the web site in 25.8% (German web projects: 53.8%) of the web sites. Some web sites, however, made use of update scripts which automatically set the ‘last update’ field to the current date (Table 4).

### Analysis of the web site content

Evaluating the content of the web sites, we found that information about the four main therapy modalities for breast cancer (surgery, chemotherapy, radiotherapy, hormone therapy) were more frequently seen on English-language web projects compared to German-language web projects (Table 4). We observed the same when we examined the presentation of new approaches such as immunotherapy (15.9 *vs* 12.3%) and stem cell transplantation (16.7 *vs* 6.2%). Conversely, palliative therapy (4.5 *vs* 16.7%) and methods of alternative medicine (18.2 *vs* 46.2%) were significantly more often presented on German-language web projects; pain therapy obviously showed the same trend (2.3 *vs* 4.6%). Side effects and risks of therapy were the domain of English-language web projects whereas German-language web projects more often dealt with disease-related information (like risk factors for breast cancer, prognosis, genetics, epidemiology, cancer aftercare, and self-help groups).

## DISCUSSION

Recent studies confirmed that patients demand high-quality health care information (Guadagnino, 2000; LoBuono, 2000; Chen and Siu, 2001; Fogel *et al*, 2002; Forbriger and Glattes, 2003). Hence, many efforts (Table 2) were made to improve the quality of online information with moderate success (Pandolfini *et al*, 2000). Any improvement needs to be monitored by reliable and valid evaluation tools for information quality. In our study, therefore, procedures and definitions of the evaluations were validated and fixed in a protocol, including detailed instructions for training of the raters. The items of the analysis were based on published and generally accepted instruments for the evaluation of information quality. However, the set of items that most accurately describe the quality of web sites (Gordon-Murnane, 1999; Jitaru *et al*, 1999) and the procedure of the analysis (Eysenbach *et al*, 2002) itself are controversially discussed.

We found the most striking differences regarding web sites dealing with ‘complementary alternative medicine’ (CAM) (Markman, 2001). We defined CAM as ‘practices not generally recognised by the medical community as standard or conventional’, including, for example, herbal preparations, megadose vitamins, magnet therapy, spiritual healing, and meditation. Significantly more German-language web sites (46.2%) than English-language web sites (18.3%) dealt with this aspect of breast cancer treatment (Table 4). Most of the German web sites dealing with CAM contained information directed against conventional therapies. Corresponding to our data, Ernst and Schmidt (2002) judged five out of 13 web sites dealing with alternative medicine as harmful for cancer patients if the advice provided was followed. Moreover, three out of these 13 web sites overtly discouraged cancer patients to employ conventional therapies.

The lower frequency of English-language web sites with alternative medicine compared to German-language web sites may be due to the legal practices in the US to hold content providers responsible for what can be regarded as ‘Internet fraud’, leading to high indemnities (Mack, 2001). Further tragic instances where misinformation brought death (Hainer *et al*, 2000) may provoke that, in future, ‘compensation for misinformation’ will be justifiable. Besides this judicial aspect, we assume differences in the needs for and susceptibility to alternative medicine between the audience of English- and German-language web sites. Anti-science attitudes meshed with New Age mysticism, belief in the superiority of ‘natural’ products, and inability to make an informed choice about a health care product (Beyerstein, 2001) were known to promote the popularity of alternative medicine, even or especially among otherwise intelligent patients (who are the typical ‘health seekers’ on the Internet). In all, 51.6% of the patients in England, however, use CAMs (Werneke *et al*, 2004), and the impact of the Internet on self-medication with herbal remedies and even on trafficking in controlled drugs is still on the rise (International Narcotics Control Board (INCB), 2004).

In general, we found information related to curative therapy (especially side effects and risks of therapy) and scientific-based aspects of breast cancer (e.g. current trials or results of recently published studies) significantly more frequently on English-language web projects. Conversely, we found information related to palliative therapy and disease-related information significantly more frequently on German-language web projects (Table 4). As most English-language web projects were of US origin, this may reflect the role of judiciary as well as the American superiority in terms of research and basic science. Apparently, the emphasis on therapy-associated risks and side effects also aim to hedge the risks of possible indemnities (similar to our finding that on English-language web sites alternative medicine is frequently disregarded, as described above).

Further, authors of English-language web projects obviously assume that their audience expects information about cutting edge therapies and studies evaluating new agents, though this information jeopardises the intelligibility and readability of their web sites (but may increase authority and credibility in the perception of the Internet users). On the contrary, the authors of German-language web projects seem to assume that their audience prefers the digest of such information rather than details about current trials or recently published studies. In fact, asking 213 patients for the most important detail of information they want to receive, we found that patients most frequently (more than 90%) answered ‘prognosis’ (unpublished data).

Similar to the US American audience, however, a recent study reported a change in access to information and patient behaviour over the past few years: Patients increasingly demand information on current clinical studies, new agents, genetic risk factors, diagnosis, and screening procedures (Hiller, 2004). These data base on 210 000 calls, e-mails, and letters, addressed to the German Cancer Information Service (German Cancer Research Center) since 1986.

Information about palliative therapy options (e.g. bisphosphonates administration or radiation of metastatic lesions) was more frequently found on German-language web projects (16.9%) compared to English-language web projects (4.6%), suggesting that English-language web projects more often focus on curative therapies (Weissenberger, 2004). Both English- and German-language web projects rarely mentioned pain management for metastatic breast cancer (English *vs* German web sites: 2.3 and 4.6%, respectively). Evidently, the majority of English- and German-language web sites focused on the curability of breast cancer, but neglected helpful information for a considerable number of breast cancer patients with metastatic disease.

Compared to German web projects, the authors of English-language web projects were convinced (if at all) of the necessity rather to ensure privacy than to disclose authorship. We found a privacy statement on 23% of the English and 14% of the German web sites. In all, 30.3% of the English web sites (German web sites: 55.4%) provided information about the authors, and 22.7% (German web sites: 49.2%) about their qualifications. Shon and Musen (1999) had found authorship and qualification statements in 20% of the medical web sites analysed (*n*=97). After 3 years, Meric *et al* (2002) found evidence of authorship in 57% of the web sites analysed (*n*=184), but only 17% displayed the author's name, qualifications, and affiliation.

In conclusion, analysing and comparing large English- and German-language breast cancer web sites, we found significant differences in web content and quality. English-language web sites provided better overall quality (often supported by scientific-based information, e.g. about current trials or new agents), but focused on curative treatment options and their risks and side effects. German-language web sites comprised significantly more often palliative and alternative medicine, but a substantial number of web sites were a serious potential danger to patients. As we pointed at the strengths and weaknesses of English- and German-language web sites and observed several intercultural differences, our results may help physicians to more accurately satisfy patients' information needs and to improve patient education. If cancer patients use the Internet for information and support needs that are not met through conventional health care (Ziebland *et al*, 2004), physicians should make efforts to correct these communicating deficiencies and provide their patients even these information that are not met through the Internet.

## References

[bib1] Achenbach J (1996) Reality check: you can't believe everything you read, but you'd better believe this. Washington Post C1 (December 4, 1996)

[bib2] Argus Associates. Ratings System (2002) http://www.clearinghouse.net/ratings.html (accessed March 9, 2004)

[bib3] Beyerstein BL (2001) Alternative medicine and common errors of reasoning. Acad Med 76: 230–237 11242572 10.1097/00001888-200103000-00009

[bib4] Bichakjian CK, Schwartz JL, Wang TS, Hall JM, Johnson TM, Biermann JS (2002) Melanoma information on the Internet: often incomplete – a public health opportunity? J Clin Oncol 20: 134–141 11773162 10.1200/JCO.2002.20.1.134

[bib5] Biel M (1997) ‘I want to stop preconceptions’ (‘Ich will aufräumen mit Vorurteilen’). Stern Magazine (February 27, 1997)

[bib6] Blanchard D, Erblich J, Montgomery GH, Bovbjerg DH (2002) Read all about it: the over-representation of breast cancer in popular magazines. Prev Med 35: 343–348 12453711 10.1006/pmed.2002.1088

[bib7] Charnock D, Shepperd S, Needham G, Gann R (1999) DISCERN: an instrument for judging the quality of written consumer health information on treatment choices. J Epidemiol Community Health 53: 105–111 10396471 10.1136/jech.53.2.105 PMC1756830

[bib8] Chen X, Siu LL (2001) Impact of the media and the internet on oncology: survey of cancer patients and oncologists in Canada. J Clin Oncol 19: 4291–4297 11731511 10.1200/JCO.2001.19.23.4291

[bib9] Ciolek TM, Goltz IM (2001) Information Quality WWW Virtual Library (The Internet Guide to Construction of Quality Online Resources)

[bib10] Cluzeau F, Burgers J (2002) The AGREE Collaboration. Appraisal of Guidelines for Research & Evaluation (AGREE) Instrument.

[bib11] Cohen JA (1960) A coefficient of agreement for nominal scales. Educ Psych Meas 20: 37–46

[bib12] Degner LF, Kristjanson LJ, Bowman D, Sloan JA, Carriere KC, O'Neil J, Bilodeau B, Watson P, Mueller B (1997) Information needs and decisional preferences in women with breast cancer. JAMA 277: 1485–1492 9145723

[bib13] Ernst E, Schmidt K (2002) ‘Alternative’ cancer cures via the Internet? Br J Cancer 87: 479–480, doi:10.1038/sj.bjc.6600513 12189540 10.1038/sj.bjc.6600513 PMC2376143

[bib14] Eysenbach G, Powell J, Kuss O, Sa ER (2002) Empirical studies assessing the quality of health information for consumers on the world wide web: a systematic review. JAMA 287: 2691–2700 12020305 10.1001/jama.287.20.2691

[bib15] Fogel J, Albert SM, Schnabel F, Ditkoff BA, Neugut AI (2002) Use of the Internet by Women with Breast Cancer. JMIR 4: e9 http://www.jmir.org; http://www.jmir.org/2002/2/e9, (accessed March 9, 2004) 12554556 10.2196/jmir.4.2.e9 PMC1761930

[bib16] Forbriger A, Glattes M (2003) The Internet through the eye of breastcancer patients and their families – or the dilemma of credible and usable websites and the patients' needs. http://www.mednet2002.org/abstracts/display.cfm?id=10513211 (accessed March 9, 2004)

[bib17] Gordon-Murnane L (1999) Evaluating net evaluators. Searcher 7(2): 57–66

[bib18] Greer S (2000) Fighting spirit in patients with cancer. Lancet 355: 847–848 10.1016/S0140-6736(05)72464-8 10711956

[bib19] Guadagnino C (2000) Using medical information on the Internet. Physician's News Digest. http://www.physiciansnews.com/spotlight/700.html (accessed March 9, 2004)

[bib20] Hainer MI, Tsai N, Komura ST, Chiu CL (2000) Fatal hepatorenal failure associated with hydrazine sulfate. Ann Intern Med 133: 877–880 11103057 10.7326/0003-4819-133-11-200012050-00011

[bib21] Health On the Net (HON) (2002) HON Code of Conduct (HONcode) for medical and health web sites. http://www.hon.ch/HONcode (accessed March 9, 2004)

[bib22] Hiller B (2004) Public perception of cancer risk – an evaluation of requests to KID, the German cancer information service. German Cancer Congress 2004 (February 27, 2004) http://www.kukm.de/krebskongress2004/abstracts/PO794.pdf (accessed March 9, 2004)

[bib23] International Narcotics Control Board (INCB) (2004) Cyber trafficking in controlled drugs on the rise. http://www.incb.org (accessed March 9, 2004)

[bib24] Jitaru E, Moisil I, Jitaru M-C (1999) Criteria for evaluating the quality of health related sites on Internet. MEDINF 2000 – The 23rd National Conference on Medical Informatics, Telemedicine and Telematics (October 15, 1999)

[bib25] Kögel M, Longo P (2001) Trendletters NFO Europe/Infratest. http://www.nfoeurope.com (accessed March 9, 2004)

[bib26] Kunst H, Groot D, Latthe PM, Latthe M, Khan KS (2002) Accuracy of information on apparently credible websites: survey of five common health topics. BMJ 324: 581–582 11884323 10.1136/bmj.324.7337.581 PMC78996

[bib27] LoBuono C (2000) Using the Internet and e-mail in your practice. Patient Care (December 30, 2000) http://www.findarticles.com/cf_0/m3233/24_34/68743460/print.jhtml (accessed March 9, 2004)

[bib28] Mack B (2001) ‘Operation cure. All’ wages new battle in ongoing – war against Internet health fraud. http://www.ftc.gov/opa/2001/06/cureall.htm (accessed March 9, 2004)

[bib29] Markman M (2001) Interactions between academic oncology and alternative/complementary/integrative medicine: complex but necessary. J Clin Oncol 19: 52S–53S 11560972

[bib30] McKenzie BC (1996) Medicine and the Internet. New York: Oxford University Press Inc

[bib31] Meric F, Bernstam EV, Mirza NQ, Musen MA (2002) Breast cancer on the world wide web: cross sectional survey of quality of information and popularity of web sites. BMJ 324(7227): 577–581 11884322 10.1136/bmj.324.7337.577 PMC78995

[bib32] Nicholson A (1996) Diet and the prevention and treatment of breast cancer. Altern Ther Health Med 2: 32–38 8942042

[bib33] NOAH (2002) Choosing the links: how NOAH (New York Online Access to Health) selects material. http://www.noah-health.org/english/collection.html (accessed March 9, 2004)

[bib34] OMNI (2002) OMNI guidelines for resource evaluation. http://omni.nott.ac.uk/agec/archive-evalguid.html (accessed March 9, 2004)

[bib35] Pandolfini C, Impicciatore P, Bonati M (2000) Parents on the web: risks for quality management of cough in children. Pediatrics 105: e1 10617738 10.1542/peds.105.1.e1

[bib36] Pichler N, Gilg HJ (2001) Women capture the Internet: more than five million women are online Germany (Frauen erobern das Internet: Erstmals mehr als fünf Millionen Frauen in Deutschland online). http://de.netvalue.com/presse/index_frame.htm?fichier=cp0025.htm (accessed March 9, 2004)

[bib37] Pimienta D, Lamey B, Prado D, Sztrum M (2001) The fifth study on languages and the Internet (Networks and Development Foundation). http://funredes.org/LC/english/L5 (accessed June 30, 2004)

[bib38] Prümel U, Philippsen J, Iseringhausen O (2002) Quality assurance for health care information systems: AFGIS, the German Action Forum Health Information System, an initiative of the German Ministry of Health. J Cancer Res Clin Oncol 128(Suppl 1) (25th German Cancer Congress): O836no

[bib39] Schulz S, Klar R, Auhuber T, Schrader U, Koop A, Kreuz R, Oppermann R, Simm H (2001) Quality criteria of Electronic Publication in Medicine – Criteria Catalogue of GMDS-AG CBT (Qualitätskriterien für Elektronische Publikationen in der Medizin – Kriterienkatalog der GMDS-AG CBT). Inform Biometrie Epidemiol Med Biol 31: 153–166

[bib40] Shon J, Musen MA (1999) The low availability of metadata elements for evaluating the quality of medical information on the World Wide Web. Proc AMIA Symp 945–949 10566500 PMC2232512

[bib41] Silberg WM, Lundberg GD, Musacchio RA (1997) Assessing, controlling, and assuring the quality of medical information on the Internet: Caveant lector et viewor – let the reader and viewer beware. JAMA 277: 1244–1245 9103351

[bib49] Smith ED, Phillips JM, Price MM (2001) Screening and early detection among racial and ethnic minority women. Semin Oncol Nurs 17(3): 159–170 11523482 10.1053/sonu.2001.25945

[bib42] Su L, Chen H-L, Dong X (1998) Evaluation of Web-based search engines form the end-user's perspective: a pilot study (Information Access in the Global Information Economy). Inform Today (Proc 61st ASIS Annu Meet) 35: 348–361

[bib43] Taylor H, Leitman R (2001) eHealth traffic critically dependent on search engines and portals (Most of the 100 million people who look for health information online do so using a portal or search engine. Only a quarter go directly to an eHealth site.). Health Care News 1, www.harrisinteractive.com (accessed March 9, 2004)

[bib44] URAC: Quality health web sites you can trust (2002) http://websiteaccreditation.urac.org (accessed March 9, 2004)

[bib45] Weir HK, Thun MJ, Hankey BF, Ries LA, Howe HL, Wingo PA, Jemal A, Ward E, Anderson RN, Edwards BK (2003) Annual report to the nation on the status of cancer, 1975–2000, featuring the uses of surveillance data for cancer prevention and control. J Natl Cancer Inst 95: 1276–1299 12953083 10.1093/jnci/djg040

[bib46] Weissenberger C (2004) Bisphosphonates on the Internet. Bone Depeche (in press)

[bib47] Werneke U, Earl J, Seydel C, Horn O, Crichton P, Fannon D (2004) Potential health risks of complementary alternative medicines in cancer patients. Br J Cancer 90: 408–413 14735185 10.1038/sj.bjc.6601560 PMC2410154

[bib48] Ziebland S, Chapple A, Dumelow C, Evans J, Prinjha S, Rozmovits L (2004) How the internet affects patients' experience of cancer: a qualitative study. BMJ 328: 564 15001506 10.1136/bmj.328.7439.564 PMC381051

